# Modeling the Spatial Distribution and Fruiting Pattern of a Key Tree Species in a Neotropical Forest: Methodology and Potential Applications

**DOI:** 10.1371/journal.pone.0015002

**Published:** 2010-11-22

**Authors:** Damien Caillaud, Margaret C. Crofoot, Samuel V. Scarpino, Patrick A. Jansen, Carol X. Garzon-Lopez, Annemarie J. S. Winkelhagen, Stephanie A. Bohlman, Peter D. Walsh

**Affiliations:** 1 Section of Integrative Biology, University of Texas at Austin, Austin, Texas, United States of America; 2 Department of Primatology, Max Planck Institute for Evolutionary Anthropology, Leipzig, Germany; 3 Smithsonian Tropical Research Institute, Panamá, República de Panamá; 4 Department of Migration and Immuno-ecology, Max Planck Institute for Ornithology, Radolfzell, Germany; 5 Department of Ecology and Evolutionary Biology, Princeton University, Princeton, New Jersey, United States of America; 6 Forest Ecology and Forest Management Group, Wageningen University, Wageningen, The Netherlands; 7 Community and Conservation Ecology Group, University of Groningen, Haren, The Netherlands; 8 VaccinApe, Bethesda, Maryland, United States of America; University of Western Ontario, Canada

## Abstract

**Background:**

The movement patterns of wild animals depend crucially on the spatial and temporal availability of resources in their habitat. To date, most attempts to model this relationship were forced to rely on simplified assumptions about the spatiotemporal distribution of food resources. Here we demonstrate how advances in statistics permit the combination of sparse ground sampling with remote sensing imagery to generate biological relevant, spatially and temporally explicit distributions of food resources. We illustrate our procedure by creating a detailed simulation model of fruit production patterns for *Dipteryx oleifera*, a keystone tree species, on Barro Colorado Island (BCI), Panama.

**Methodology and Principal Findings:**

Aerial photographs providing GPS positions for large, canopy trees, the complete census of a 50-ha and 25-ha area, diameter at breast height data from haphazardly sampled trees and long-term phenology data from six trees were used to fit 1) a point process model of tree spatial distribution and 2) a generalized linear mixed-effect model of temporal variation of fruit production. The fitted parameters from these models are then used to create a stochastic simulation model which incorporates spatio-temporal variations of *D. oleifera* fruit availability on BCI.

**Conclusions and Significance:**

We present a framework that can provide a statistical characterization of the habitat that can be included in agent-based models of animal movements. When environmental heterogeneity cannot be exhaustively mapped, this approach can be a powerful alternative. The results of our model on the spatio-temporal variation in *D. oleifera* fruit availability will be used to understand behavioral and movement patterns of several species on BCI.

## Introduction

How animals use their habitat has important consequences for their resilience to anthropogenic perturbations [1], [2], [3], [4], [5], susceptibility to diseases and ability to provide critical ecosystem services (*e.g.* pollination and seed dispersal—see refs [6], [7], [8], [9], [10]). Although many factors impact patterns of animal movement, variation in the spatiotemporal distribution of food resources plays a critical role [11], [12], [13], [14]. As a result, a growing body of research has focused on linking animal movement patterns and ecological variables (*e.g.*, refs [15], [16]).

Obtaining fine-scale movement data has become possible thanks to recent advances in automated radio telemetry and GPS tracking technology (*e.g.*, refs [17], [18]). The large datasets produced by these methods, with their high temporal and spatial resolution, provide an unprecedented opportunity for understanding the psychological or physiological states and decision mechanisms that govern animal movement. A critical component of these models is a representation of the spatial and temporal distribution of key ecological variables. In some cases, as for species living in open habitats where the scale of environmental heterogeneity is relatively coarse, researchers have been able to build powerful inferential movement models [4], [9], [19], [20]. However, it remains a challenge to model the ecological component when the spatial and temporal distributions of resources are finer grained, or temporally variable. This is true for animals relying on marine food resource, like seabirds [1], [21] or marine fish [13], but is also the case for animals that move on a large scale but exploit relatively fine-grained habitat variation, such as tropical forest-dwelling frugivores. In tropical forests with hundreds of tree species, it is rarely feasible to directly map all possible food sources, and remote sensing imagery is generally unable to identify individual fruit tree species or to score whether each tree is in fruit (but see ref [22]). In these cases, the traditional statistical inference approach, which models movement data as a function of resource abundance at each spatial location of an animal's range, fails. Stochastic, agent-based modeling may provide a solution to this puzzle.

Agent-based models attempt to reproduce the *statistical* properties of observed movement data. As a result the simulated habitat maps which serve as the background for such models need not perfectly match the actual distribution of resources; they must simply reproduce the inherent statistical properties of the real system. Thus, the challenge when using an agent-based modeling approach is not how to exhaustively map a study animal's habitat but, instead, how to statistically characterize the inherent properties of that habitat and incorporate them into a model that generates realistic resource distributions. Importantly, such a model can be created using various sources of information, such as satellite or aerial imagery, ground cartographic surveys and phenological surveys. These data do not need to be collected at the same time or place, nor do they necessarily need to be collected in the range of the monitored animals. The only requirement is that they should be collected in a habitat with a similar pattern of resource spatial distribution.

Here we use data from Barro Colorado Island (BCI), Panama, to demonstrate how a variety of ground surveys and remote sensing data can be combined to create the kind of robust habitat model necessary for agent-based simulations of animal movement. We first combine systematic plot data and aerial survey data to estimate density, size distribution, and spatial autocorrelation for *Dipteryx oleifera* Benth (Fabaceae), a fruit tree species that acts as an important food resource for several large mammals on BCI [23]. We then use 22 years of phenological data from fallen fruit traps to estimate the fruiting pattern of *D. oleifera*. These two approaches were merged to create a spatially and temporally-explicit model of *D. oleifera* fruit distribution of BCI.

## Analysis

### 1. Data collected

#### Study Site and Species

Barro Colorado Island (BCI), Panama (9°9′ N, 79°51′ W) is a 15.6-km^2^ island of semi-deciduous lowland tropical forest that was isolated from mainland Panama in 1914 when the Chagres River was dammed to form Lake Gatun and the Panama Canal. Designated a reserve in 1923, BCI has been administered by the Smithsonian Institute since 1948. Half of BCI is covered by relatively young forest (at least 100 years old) that is still growing back from clearing that occurred during the French attempt to build the canal in the late-1800s. The remainder of the forest is older, and is not thought to have undergone substantial anthropogenic disturbance in the last 200–400 years [24]. This older forest is quite diverse, containing 299 tree species in a 50-ha plot [24].

Patterns of rainfall on BCI are distinctly seasonal; the island receives an average of 2600 mm of rainfall a year, 90% of which falls between May and December [25]. Food availability for primary consumers roughly tracks these changes in rainfall. Fruit and leaf production are highest during the late dry season and early wet season, while the late wet season (October and November) is a period of food scarcity [26], [27]. Few trees fruit or flush leaves during these months and, in extreme years, this lack of food resources can lead to mass starvation among the vertebrates [24]. This period of scarcity is broken by the fruiting of *D. oleifera* at the start of the dry season (mid-December).

*Dipteryx oleifera*, formerly *Dipteryx panamensis*, is a large emergent tree (40–50 m), ranging from Costa Rica to Colombia. *D. oleifera* produces large (average length = 5 cm; average fresh weight = 21.75 g, Crofoot unpublished data), sugar-rich drupes containing a single 4-cm-long seed surrounded by a hard endocarp [23]. Fruiting phenology is variable between years and individuals, and typically lasts from late December until early April [23], [27]. Seeds are primarily dispersed by frugivorous bats and secondarily dispersed by scatter-hoarding rodents, and heavily predated upon by terrestrial mammals [28], [29], [30]. In Central Panama, *D. oleifera* is the focus of frugivore activity for up to 2.5 months [31] and attracts a wide range of animals including bats (*Artibeus jamaicensis*), kinkajous (*Poto flavus*), squirrels (*Sciurus granatensis*), spiny rats (*Proechimys semispinosus*), monkeys (*Cebus capucinus*, *Ateles geoffroyi*), coatis (*Nasua narica*), agoutis (*Dasyprocta punctata*), pacas (*Agouti paca*), peccaries (*Tayassu tajacu*), deer (*Odocoileus virginianus*) and tapir (*Tapirus bairdii*).

#### Spatial data

**Figure 1:**
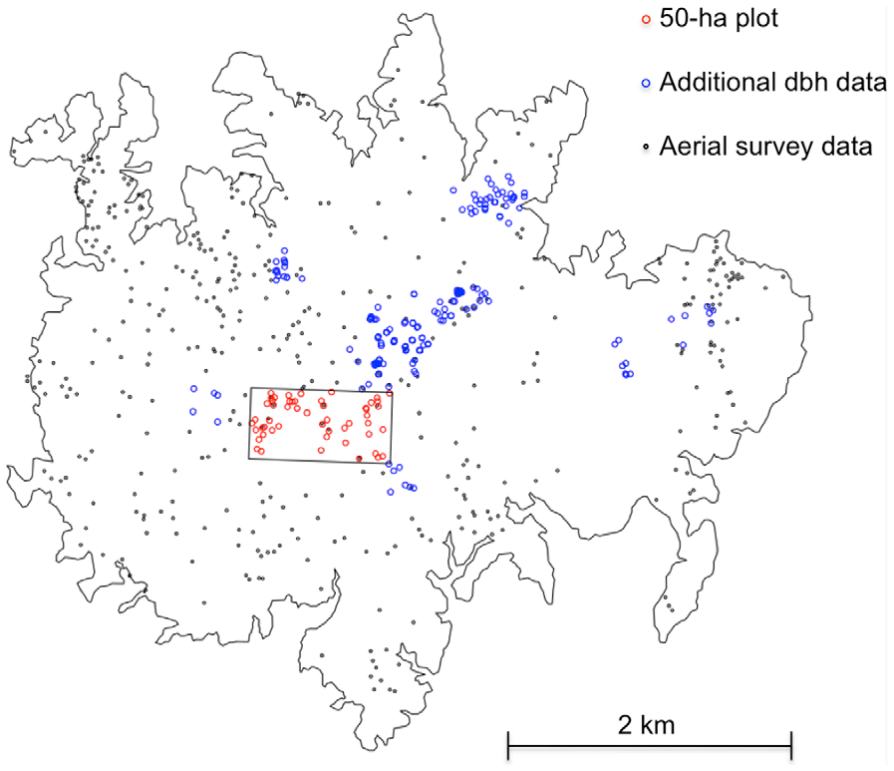
Location of individual *Dipteryx oleifera* included in this study. The additional DBH data include the 50-ha plot, the 25-ha plot and the home-range based *D. oleifera* plots.

Four spatial datasets were used to estimate the tree size distribution of *Dipteryx oleifera* on BCI and to model the spatial distribution of individual trees. (1) Mapped stem positions and diameters in a 50-ha forest dynamics plot, established in old-growth forest on the central plateau in 1980 (see Fig. 1), in which every stem over 10 mm DBH (Diameter at Breast Height) was mapped, measured and identified [32], [33], [34]. This plot is censused every five years, and the resulting dataset has been made freely available (http://ctfs.arnarb.harvard.edu/webatlas/datasets/bci/). (2) Mapped stem positions and diameters in an additional 25-ha plot established in 2004, where all trees >20 cm DBH and all reproductive individuals from large-seeded species (seed weight >1 gram) were mapped, measured and identified. The 25-ha plot is located in secondary forest, estimated to be 100–120 years old [22]. (3) Mapped stem positions of 102 individuals >30 cm DBH across the home ranges of 11 agoutis (*Dasyprocta punctata*), surveyed in 2009. Trees were mapped using a handheld GPS (Garmin GPSMap 60CSX, Garmin International, Inc., Olathe, KS). This sampling area does not have a clearly defined border (4) Mapped positions and area of crowns of canopy-statured individuals across the entire BCI, obtained from aerial surveys in April 2005 and April 2006. High-resolution (0.085–0.114 meters/pixel) aerial photographs were taken with a 12.3 megapixel digital SLR camera (Fuji FinePix S3 Pro with a 35 mm lens, f-stop 4.5–4.8, shutter speed 1/700–1/1000 s, and ISO speed 400) from an airplane flying at either 400 meters (2005) or 700 meters (2006) above the canopy [22]. In 2005, each photo on average covered 8.6 ha (358×241 m) with a spatial resolution of 0.085 m/pixel. In 2006, coverage and resolution averaged 15.9 ha (483×329 m) and 0.114 m/pixel. [22]. Two different analysts visually surveyed the georeferenced aerial photographs for *D. oleifera*, which they identified based on canopy structure (C.X. Garzon-Lopez, unpubl. data).

#### Phenology data

Fruits fall from trees following a consistent vertical trajectory [35], allowing researchers to estimate fruit productivity by counting the fruits found below focal trees. Within the 50-ha forest plot, three hundred 0.5-m^2^ fruit fall traps have been arrayed since 1987 [36], [37]. Traps are checked weekly, and all reproductive plant parts found are counted and identified to species. A total of 22 years of data (1987–2008) were kindly made available to us by Dr. S. Joseph Wright (http://striweb.si.edu/esp/meta_data/index_metadata_terr.htm). Ten of the phenology traps within the 50-ha forest dynamics plot are located underneath the crowns of 6 different *D. oleifera* trees. The number of fruits collected under a single tree during a visit varied between 0 and 15. In a given fruiting season, up to 52 fruits were collected under a tree (mean = 7.14, sd = 10.45). A total of 943 fruits were trapped during this study.

### 2. Model design and fitting procedure

To build a spatiotemporal model of *D. oleifera* fruiting we split the problem in to two parts: first, modeling the spatial distribution of *D. oleifera* and second modeling its fruiting pattern. These two models could then be combined and used to simulate data with spatial and temporal distribution of fruit matching those observed on Barro Colorado Island.

#### Analysis of tree spatial distribution

In our model of spatial distribution, trees are discrete entities represented as points distributed in a two-dimensional space following a random process, a type of graphical representation know as a spatial point pattern [38], [39], [40]. A variety of point process models exist that can be fit to point patterns. These models include parameters that account for environmental covariates and interactions between neighboring points. Outputs of fitted models can subsequently be used to perform Monte Carlo simulations of spatial point distributions.

The basic reference model of a point process is the homogeneous Poisson process, which assumes that the points are distributed randomly and independently in the environment. The number of points falling in any given area *A* thus follows a Poisson distribution with parameter *λ.A*, in which *λ* is the intensity of the process (*i.e.*, the point density). Such a model can be fit using a maximum likelihood method [41].

**Figure 2:**
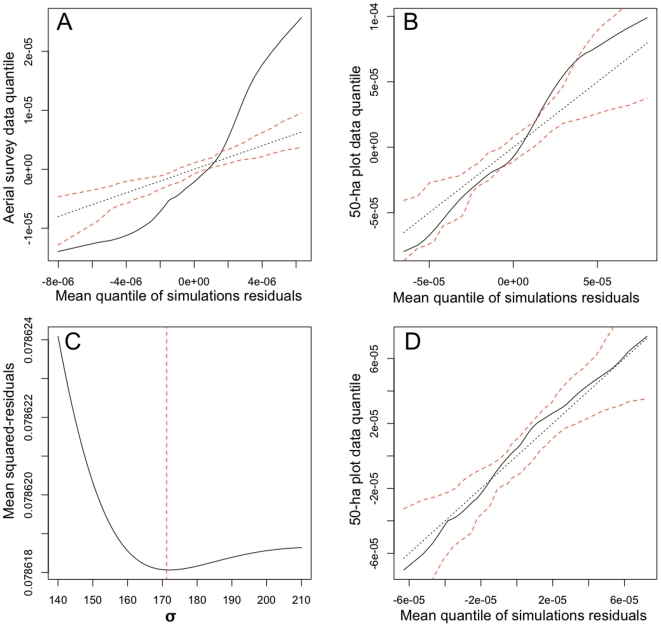
Goodness-of-fit of the Poisson point process models. A, B) Quantile-quantile plots (qq-plot) of the residuals of the homogeneous Poisson models for both datasets in relation to the mean quantiles of 100 simulations of the fitted models (solid line), with 95% critical envelopes (dashed red line). C) Optimization of the *σ* parameter of the kernel isotropic Gaussian function (minimum value indicated by the dashed red line). D) qq-plot of the inhomogeneous Poisson model including kernel-estimated aerial-surveyed-trees density as a covariate, fitted to the 50-ha plot data. The solid line lies within the 95% critical envelope, indicating a satisfying goodness-of fit of the model.

We fitted this model to the two datasets corresponding to areas that were large enough and clearly delimited: the aerial survey and a subset of the 50-ha plot including all *D. oleifera* with DBH>200mm. The model provided a poor fit to the data in both cases (see fig. 2a–b). We relaxed the homogeneity assumption of the model by fitting the 50-ha plot data to an inhomogeneous Poisson point process model in which *λ* co-varies with a spatial variable. After comparing the aerial survey and the data collected on the ground, it was apparent that only a subset of the total trees were visible in the aerial survey data (14.7% of the trees with DBH>200 mm). The probability 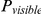 of a ground-surveyed tree being visible in the aerial survey was found to be positively correlated with DBH (logistic generalized linear model, log-likelihood ratio test,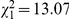, *P*<10^−3^) and can be described by the following equation:
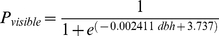
(1)


**Figure 3:**
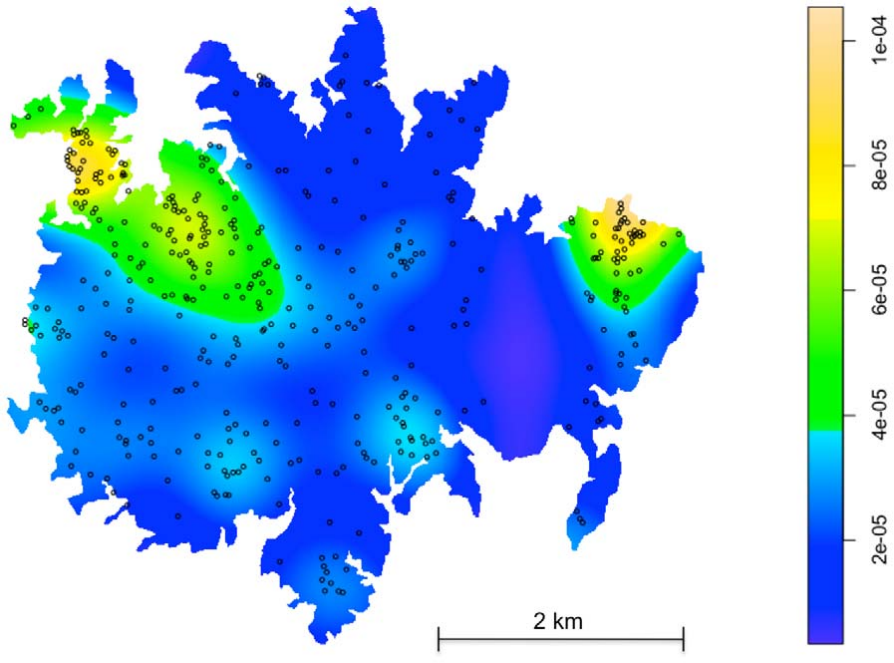
Density of the *Dipteryx oleifera* from the aerial survey data (in trees/m^2^). Only an estimated 14.7% of all trees are visible on this survey.

The density of aerial-surveyed trees may then be a good proxy for the overall tree density and was chosen as a spatial covariate. The density of trees for the whole island was thus computed by applying an isotropic Gaussian smoothing kernel function to the aerial survey data augmented with the probability statement in equation (1) (fig. 3). The standard deviation, *σ*, of the kernel function was chosen in order to optimize the fit of the model (fig. 2c). The best model, obtained for *σ* = 172 m, displays a satisfying goodness-of-fit (fig. 2d).

**Figure 4:**
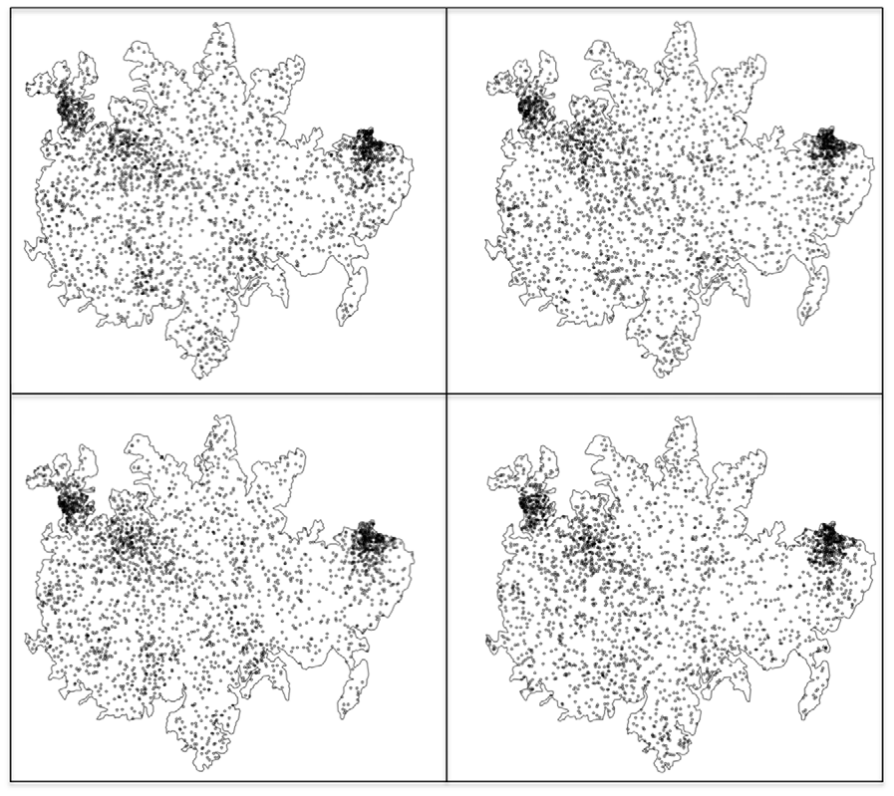
Monte-Carlo simulation of spatial distributions of *Dipteryx oleifera*. Note that none of the real, sampled trees are included.

Subsequently, the fitted values of our inhomogeneous Poisson model were used in conjunction with the values of the tree density covariate to simulate tree distributions for the entire island (see examples on fig. 4). The DBH's of these trees were drawn from a distribution matching closely the real distribution of the DBH's of the ground-surveyed trees (50-ha plot, 25-ha plot, agouti home-range based plot) using a rank-transformation to normality.

The point process models were fit using the packages *spatstat* and *maptools*, run on R 2.10.1.

#### Analysis of tree fruiting patterns

To construct a model of *D. oleifera* fruiting patterns that captures the relevant statistical properties of the system, we needed to consider the variation in fruit production pattern observed within and between both seasons and trees. In particular, we focused on the variation of three phenological traits: the fruit production, defined as total number of fruits trapped; the fruiting peak, defined as the mean of the time (expressed in days since previous July 1^st^) at which the fruits were trapped; and the duration of the fruiting period, defined as four times the standard deviation of the time at which the fruits were trapped. Values of these three metrics were computed for each of the 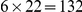 tree-season combinations.

**Figure 5:**
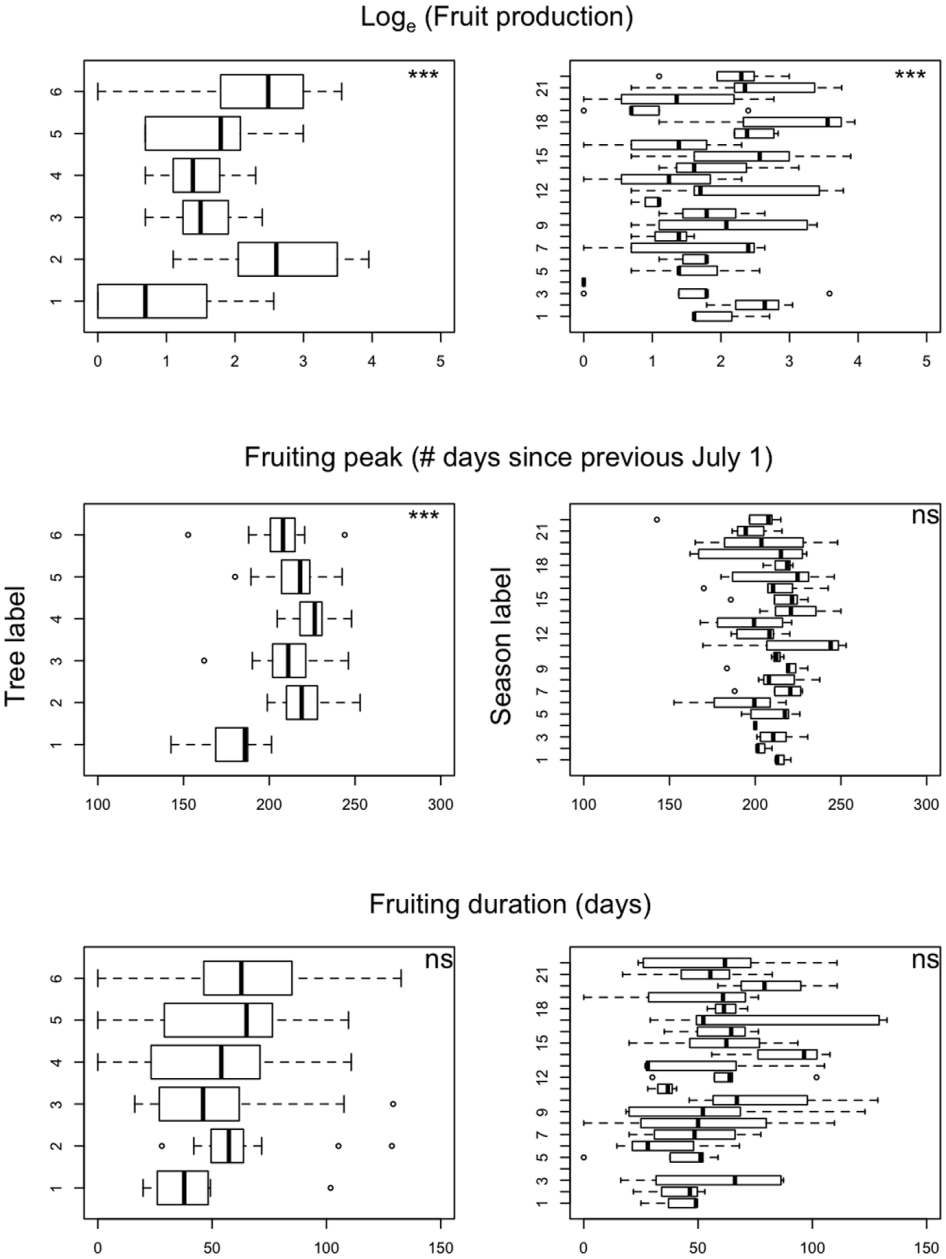
Distributions of the fruit production, fruiting peak and fruiting duration among trees (left, N = 6) and seasons (right, N = 22). See definitions in main text.

We considered the fruiting seasons and the individual trees as sampled at random from the very large population of possible seasons and tree individuals. This consideration permitted us to treat the season and the tree as two random effects variables (“season id” and “tree id”, respectively) in a mixed-effects model. As opposed to fixed-effects models, mixed-effects models do not estimate average deviations observed for each level of random effect variables, but instead estimate the variance of these deviations. For example, if one considers a model with a single random variable containing n = 10 levels, then n−1 = 9 parameters (*i.e.*, the nine deviations) are estimated with a fixed-effects model, while only one parameter (*i.e.*, the variance) is estimated with a mixed-effects model. This has two main advantages. First, it saves degrees of freedom, and hence generally reduces the variance of the estimators of the fixed effects, and second, some variance parameters can be estimated, which in the case of our model are more relevant descriptors of the biological phenomena. Indeed, we are interested in understanding between-tree and between-season variability in fruiting patterns, rather than the deviations associated with particular trees or seasons. The box plots in figure 5 display the variation of the distribution for the three metrics described above, as they occur between trees and between season.

Three linear mixed-effects models (LMEM) with tree identity and season identity as crossed random effects were fit to the datasets corresponding to each of the three metrics described above (see fig. 5). No fixed-effect variables were included in the fruiting-peak and fruiting-period models, while the fruit-production model included the tree diameter at breast height (DBH, in millimeters) as a quantitative, fixed-effect variable. Since fruit amounts are counts, the LMEM used for this dependent variable was a generalized LMEM with Poisson error and Log link-function. The two other LMEM's had Gaussian errors and identity link-functions. All LMEM's were fit using the restricted maximum likelihood method implemented in the lme4 package of R 2.10.1.

**Figure 6:**
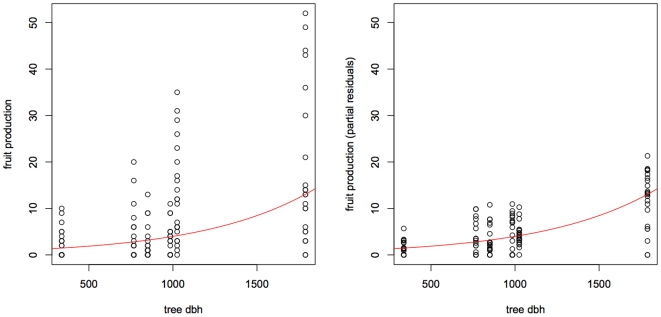
Relationship between tree DBH and fruit production. Left: raw data. Right: Row data corrected for the best predictors of the random effects.

The random effect associated to “tree id” was significant in the fruit-production and fruiting-peak models (fruit-production, sd = 0.49, p<10^−3^; fruiting-peak: sd = 15.49, p<10^−3^; fruiting-period: sd = , 1.74, p = 0.40), while the random effect associated to “season id” was significant only for the fruit production model (fruit-production : sd = 0.87, p<10^−3^; fruiting-peak: sd = 5.04, p = 0.12; fruiting-period: sd = 1.94, p = 0.44). As expected, the DBH had a significant, positive effect on fruit production (intercept *c_1_* = −.12, coefficient *c_2_* = 1.50 10^−3^, p = 0.033, see fig. 6). Intercepts for the fruiting-peak and fruiting–period models were estimated to 209.3 days and 59.3 days, respectively. All p-values were based on 1000 bootstraps.

**Figure 7:**
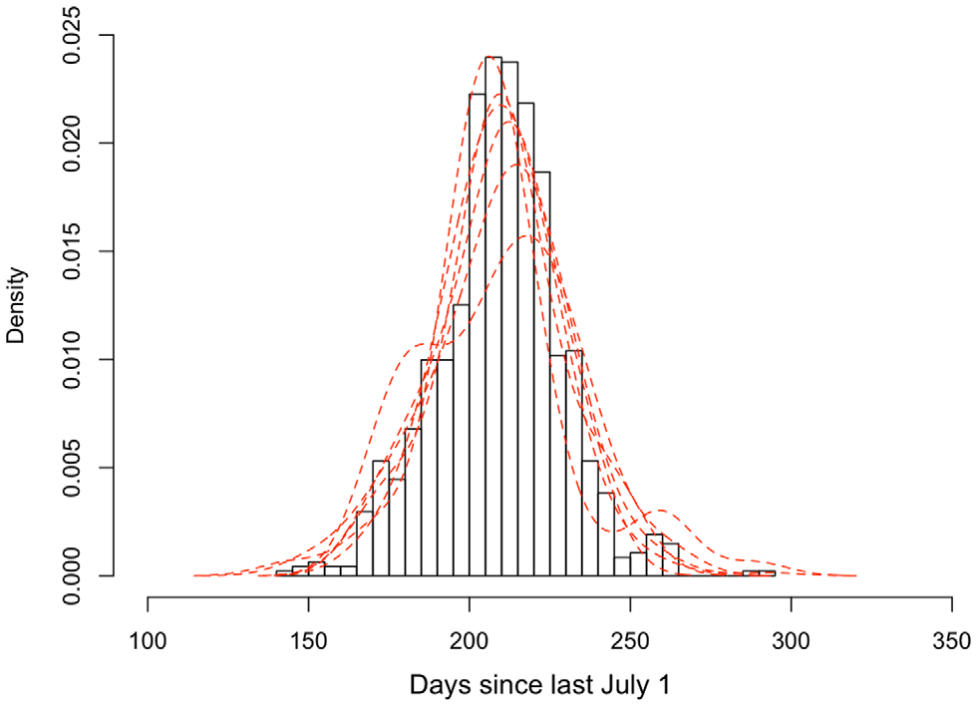
Distribution of the fruit production during the course of the year. Barplot: All data pooled together. The red dashed lines are density functions obtained for the six trees (using Gaussian kernels with sd = 10). The x variable is corrected for the tree and season effects on the fruiting peak.

Within fruiting seasons, the production of fruit trees follows a symmetric bell shape-curve (fig. 7). This observation suggests fitting the amount of fruit trapped each week under a given tree using a generalized non-linear mixed-effects model with Poisson error as an intuitive approach. For example, a possible non-linear function of fruit per unit time would be the Gaussian function:
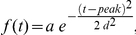
with *a*: height of the peak of the fruit production, *peak*: fruiting peak and *d*: measure of the duration of the fruiting period. In this example, *a* and *peak* would vary randomly (and independently) with tree and season identity (crossed-random effects). Unfortunately, the non-linearity of this model renders its computational fitting process prohibitively intensive and prone to estimation errors (Caillaud and Scarpino, unpublished results).

Therefore, we propose an alternative procedure combining two mixed-effect models, which allows achieving exactly the same task much more rapidly and reliably. It is important to consider that this procedure follows naturally from the intuition in the aforementioned Gaussian model. The first model (model I) aims to explain the total number of fruit trapped under each tree during an entire fruiting season. It corresponds to the fruit-production generalized LMEM described above. The second model (model II) aims to explain the date when each fruit was trapped. It is a linear mixed-effects model with normal error distribution and identity link function and includes tree identity and season identity as crossed random effects. It estimates three variance parameters: the between-tree variance, the between-season variance, and the within-tree, within-season variance. The key feature of this approach is that the latter variance (*e.g.*, the residual variance) quantifies the duration of the fruiting season, in lieu of the parameter *d* of the above equation. Therefore these two models provide results that are equivalent to the non-linear model presented above, but since they are linear models they can be fitted more rapidly and with higher reliability.

The between-seasons, between-trees and within-season standard deviations were estimated to be 8.51, 13.31 and 19.62, respectively, with intercept *c_3_* = 209.3. These parameter estimates were then used to generate fruiting patterns of simulated sets of *n* trees during *s* seasons. We proceeded in two steps.

1. We simulated two vectors ***u*** and ***v*** of respective size *n* and *s* corresponding to the respective tree identity and season identity effects on the fruit production. These values were drawn from normal distributions with mean 0 and variances identical to the estimates of the random effects from model I. The expected number of fruits produced and trapped for each of the 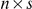 tree-season combinations were then simply computed as:
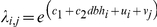
with *i* and *j* denoting the respective indices of the tree and the season, and *c*_1_ and *c*_2_ denoting the fixed effect of the variable *dbh* and the associated intercept, as estimated in model I (see values given above). The exponential function accounts for the Log-link function used in model I.
2. We simulated the two vectors ***w*** and ***z*** of size *n* and *s* corresponding to the respective tree identity and season identity effects on the mean day of fruit fall (*i.e.*, the peak of the fruiting season). These values were drawn from normal distributions with mean 0 and variances identical to the estimates of the random effects from model II. The expected amount of fruits produced at time *t* by tree *i* during season *j* was then computed as:
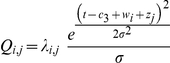
with *c*_3_: intercept (value given above) and *σ*: residual standard deviation estimated by model II. Note that the unit of 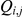 is arbitrary. The simulations were performed using R 2.10.1.

In the supplemental materials available online, we display a realization of the simulation model, run for 6 years on the same tree spatial distribution (Video S1).

## Discussion

Food resources are an important driver of animal foraging behavior and range-use patterns. Here, we show how empirical data on the spatio-temporal variation in the availability of *D. oleifera* fruit collected at a relatively small spatial scale can be combined with incomplete but larger-scale data on the spatial distribution of this species to create a habitat-wide resource availability model. We demonstrate that the distribution of this key resource is highly heterogeneous, at both spatial and temporal scales.

Our model revealed that *D. oleifera* density varied by a factor of about five across the island (fig. 3), with important clusters of trees in the northeast and northwest sectors. During the fruiting season, this heterogeneity in resources likely has an influence on the movement patterns of not only frugivorous species but those that rely on them, *e.g.* predators. Theoretical models of food searching behaviors predict that the distribution of step lengths and the distribution of the turning angles observed between steps should vary in relation to the density of food items. When a resource is scarce and patchy, distributed on spatial scale beyond the animal's sensory range, the best search strategy consists in moving according to a Levy process with step lengths following an inverse square power law distribution, while when the density of targets increases, a less skewed path length distribution (*e.g.*, Brownian motion) increases foraging efficiency [21], [42], [43].

At a temporal scale, the model shows, that fruit production reaches a maximum in January–February and lasts around 100 days. More interestingly, our model reveals that even after controlling for DBH there is still significant inter-tree variation in total fruit production and peak fruiting date. In recent theoretical work, Boyer et al. [44] and Boyer & Walsh [45] have shown that when tree distribution is random, between tree variation in fruit production can have a strong influence on the movement patterns of animals using memory of the location and size of visited trees. Therefore, we might expect animals feeding on *D. oleifera* fruit to display temporal variation in their movement patterns, both within and between years.

The impact of resource driven, temporal variation on animal movement patterns also has potential implications for disease transmission. Recent work has shown that heterogeneous resources, either in space or time, can be a major driver of inter-group contacts, often resulting in non-Euclidean distance being a better predictor of inter-group contact rates than Euclidean distance [46], [47]. These intergroup interactions, even if rare, can be important determinants in the spread of parasites and pathogens. The results of our model not only suggest that the heterogeneity of *D. oleifera* fruiting, a keystone resource on BCI, could generate the type of inter-group contacts shown to be important for disease transmission, but also provides a mechanistic explanation for their existence. The ability to understand the drivers of resource heterogeneity provides the intriguing possibility of utilizing mechanistic explanations of resource heterogeneity to design intervention strategies, e.g. targeted vaccination.

Our work demonstrates how several aspects of fruit production patterns that should be considered when studying the movements of frugivorous species can be integrated into a computationally efficient resource distribution model. Spatial distribution of trees can be investigated using spatial point pattern analyses, while inter-seasonal and inter-tree variation in fruit amounts, fruiting peak time and fruiting duration can be analyzed using mixed-effect models. These methods can also be used to generate stochastically resource distribution data using parameter values fixed a priori. Note that this framework could be easily extended to any kind of resource other than fruit, provided it has a discrete spatial distribution. Because they are not limited by the complexity of the spatial distribution of resources, agent-based simulation models appear particularly adapted to investigate animal movement using this framework. A key challenge remains however to fit these agent-based models to actual movement data. We suggest to use a set of recently developed Bayesian methods termed as Approximate Bayesian Computation (e.g., refs [48], [49], [50]). They allow circumventing the difficulty of calculating a likelihood function inherent to the more traditional maximum-likelihood or Bayesian approaches, which make them particularly appealing in our case.

## Supporting Information

Video S1 This video shows outputs of the simulation model. Fruit patterns of Dipteryx oleifera on Barro Colorado were simulated for 6 entire years (365 days), starting on July 1^st^. The six simulations use the same tree spatial distribution. Note the clear among-tree and among-season variation in fruit production. (DIVX)
